# Ecological Niche Modeling of Water Lily (*Nymphaea* L.) Species in Australia under Climate Change to Ascertain Habitat Suitability for Conservation Measures

**DOI:** 10.3390/plants11141874

**Published:** 2022-07-19

**Authors:** John M. Nzei, Virginia M. Mwanzia, Boniface K. Ngarega, Paul M. Musili, Qing-Feng Wang, Jin-Ming Chen, Zhi-Zhong Li

**Affiliations:** 1 Key Laboratory of Aquatic Botany and Watershed Ecology, Wuhan Botanical Garden, Chinese Academy of Sciences, Wuhan 430074, China; johnmulinge5@gmail.com (J.M.N.); virginiamwanzia@gmail.com (V.M.M.); bonfacebonke.bb@gmail.com (B.K.N.); jmchen@wbgcas.cn (J.-M.C.); 2 Center of Conservation Biology, Core Botanical Gardens, Chinese Academy of Sciences, Wuhan 430074, China; qfwang@wbgcas.cn; 3 University of Chinese Academy of Sciences, Beijing 100049, China; 4 East Africa Herbarium, National Museums of Kenya, Nairobi P.O. Box 451660-0100, Kenya; pmutuku@museums.or.ke; 5 Sino-Africa Joint Research Center, Chinese Academy of Sciences, Wuhan 430074, China

**Keywords:** climate change, Australia, bioclimatic variables, habitat suitability, *Nymphaea*

## Abstract

The International Panel on Climate Change (IPCC) projects a global temperature rise of 4.3 ± 0.7 °C by 2100 and an extinction of 8.5% in one out of every six species. Australia’s aquatic ecosystem is no exception; habitat loss, fragmentation, and loss of biodiversity are being experienced. As the center for *Nymphaea* species distribution, it presents the culturally, ecologically, and scientifically important genus as the best candidate for habitat suitability assessment in climate change, whose habitat suitability is presumed to decline. The models were run according to the maximum entropy (MaxEnt) method, using one general circulation model (GCM). Projections were made for the current, past, and future in medium (4.5) and high (8.5) representative concentration pathways. Significantly, bio2 and bio15 were highly preferred among the species. Less distribution was noted in West Australia compared to the north, east, and south of the continent, while north of the continent in Western Australia, Northern Territory, and Queensland indicate more habitat contractions compared to the east and southeast of Queensland and New South Wales, although it receives high precipitation. Generally, the species respond variably to both temperature and precipitation variables which is a key species response factor for planners and decision makers in species habitat and biodiversity conservation.

## 1. Introduction

Global warming is termed one of the main factors affecting world biodiversity and freshwater ecosystems. Over the past century, it has been approximated that global warming has caused the earth’s surface temperature to rise by 0.6 °C [1], and by 2100, it has been projected to increase by 4.3 ± 0.7 °C [1,2]. Climate change from the increasing temperature and anthropogenic factors are leading to the decline of freshwater ecosystems, such as 3.9% between 1970 and 2012 per year [3], and an increase of species extinction of approximately 8.5% of one in every six species to 3 °C [4]. The climatic variables consisting of annual summaries of temperature and precipitation values have shown changes in species composition and distribution in global climate change [5].

In Australia, surface temperatures are estimated to have risen by +0.94 °C over the last 100 years and by the end of the century between 2.8 and 5.1 °C [6,7] probably because of the high amount of greenhouse gas emissions over the past years [8,9]. East and South of Australia in Queensland (QLD) and New South Wales (NSW) have experienced a substantial decline in precipitation, causing the shift of periodic wet rainfall to dry. In contrast, northwest Australia has become wetter over this time, mainly during summer. In addition, a sea-level rise of approximately 17 cm in the 20th century and an average of 10 cm between 1920 and 2000 has also occurred in coastal regions [6]. As a result of climate change, climatic shifts will be experienced in many parts, especially the southeast and west of the continent. This will intensify wildfires, mostly accompanied by drought seasons, thus causing substantial impacts on water sources, coastal ecosystems, and biodiversity. Furthermore, water temperatures will rise and water salinity will increase, thus affecting the freshwater species [10,11]. For example, the waters southeast of Australia are approximated to warm at least four times the average global temperature [12]. The effects include increased mortality of aquatic species, reduced population size, changes in distribution, and shift of species to favorable habitats [10].

Freshwater and wetland ecosystems in Australia are essential and support approximately 125,500 freshwater species [13]. However, the ecosystems exhibit vulnerability to the predicaments associated with climate change [13], which leads to habitat loss and at the same time a major concern in biodiversity conservation. To evaluate the consequences of climate change on biodiversity and species habitat distribution, researchers over the past years have tried to outline its effect on species distribution, species shift, population reduction, suitable habitat change, extinction, and phenology alteration [14,15]. The studies not only lay a foundation for theoretical research but are also informative in decision making, conservation planning, and management status of species distribution. This has been made more effective by the use of methods such as species distribution models (SDMs), which have gained much attention over the years from their increasing use in understanding the historical and geographical distribution of species in climate change [16,17]. Although uncertainties have been noticed with the model predictions [18], they have been used successfully in estimating the likely impact of climate change on species distribution, shift, population decline, and possible invasion areas by invasive species [19,20], thus being incorporated in planning and management of the existing biodiversity. Their reliance has also led to constant improvement in predicting power and transferability [21].

The aquatic ecosystem in Australia is rich in species diversity and endemicity, among which *Nymphaea* species and mostly sub-genus *Anecphya* are mainly distributed. The species portray characteristic distribution, which is probably mediated by the environment. For example, some species are constrained to the continents monsoon tropics, such as *N. gigantea*, others are widespread (*N. immutabilis*, *N. macrosperma*, *N. violancea*), whereas species such as *N. hastifolia* have limited distribution [22], which increase the species vulnerability to habitat contractions or shifts under climate change [23]. In addition, the *Nymphaea* species habitat areas are precipitation dependent, with some species occurrences or populations occurring in pools replenished by flood plains water. When the temperature rises, evaporation increases, causing moisture loss, which leads to droughts and salinity of the water pools that make the habitats. Although not considered in this study, climate change and other factors such as human influence threaten some ecosystems such as coastal upland swamps in the Sydney bioregion, and seasonal herbaceous wetlands of the temperate lowland plains become endangered [24]. This poses a threat to the inhabitants of the ecosystem, necessitating our study. Although species are known to adapt to the changing environment, future climate change is likely to push the species’ adaptive limits, demanding them to shift, remain, or adapt in situ [25]. Several studies on animals, plants, insects, amphibians, reptiles, and invasive species have indicated habitat contraction or shift as a result of climate change [26,27]. Previous studies focusing on *Ottelia* and *Nymphaea* species in Africa have reported the variable change of suitable areas under climate change [28,29]. In this climate-change-prone environment, the wild populations of *Nymphaea* have declined significantly from the effects of climate change. The decline, loss of habitat, or disappearance of this species is detrimental ecologically, culturally, and scientifically, as the species are termed keystone species in aquatic ecosystem stabilization, reducing water turbidity and providing food to aquatic animals, birds, and humans [30,31].

To determine the climatic restricting factors and the habitat suitability for the *Nymphaea* species, maximum entropy (MaxEnt), an SDMs approach, was used to create distribution models for the 14 water lily species in Australia. To achieve the objective, (i) the limiting climatic factors that determine the *Nymphaea* species distribution in the continent and (ii) the past and future possible habitat suitability change were determined, hypothesizing that climate has substantially changed from the past to the future.

## 2. Results

### 2.1. Variable Selection and Performance of the Models

The potentially suitable habitats for the species were simulated using the uncorrelated climatic variables identified using Pearson correlation of value 0.7, using VIFs in the usdm R package. The VIF values were less than 10 (Table 1). The correlation analysis indicates bio2 as the main variable common to all species, followed by bio3, bio14, and bio15, and the least selected were bio5, bio6, and bio10. The majority of climatic variables supported the distribution of *N. ondinea*, *N. alba*, *N. hastifolia*, *N. georginae*, while the least for *N. carpentariae*, *N. gigantea*, *N. immutabilis*, *N. jacobsii*, *N. macrosperma*, and *N. nouchali*. Moreover, bio10 was only selected for *N. alba*. The MaxEnt features selected using ENMeval indicate that LQH and LQHP were the most common feature among the species and the least feature was LQ, with the rm feature values ranging from 0.5 to 3 (Table 2). In evaluating the suitability of the models, all AUC values ranged from greater than 0.8 to values slightly less than 1, indicating a good model performance (Table 3). In addition, the ratio between the test AUC and training AUC indicates good model performance with a value difference of less than 0.5 among the species in all scenarios (Table S1).

### 2.2. The Variable Contribution

**Figure 1:**
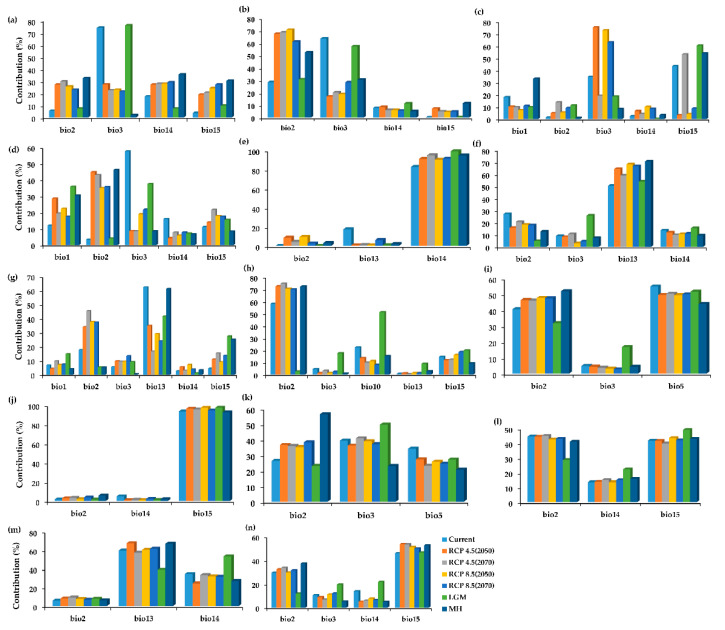
Estimates of the relative contributions of the environmental variables using MaxEnt models, fitted to the current, past, and future projections for *Nymphaea* species in Australia. (**a**) *N. atrans*, (**b**) *N. elleniae*, (**c**) *N. georginae*, (**d**) *N. hastifolia*, (**e**) *N. jacobsii*, (**f**) *N. pubescens*, (**g**) *N. ondinea*, (**h**) *N. alba*, (**i**) *N. nouchali*, (**j**) *N. carpentariae*, (**k**) *N. gigantea*, (**l**) *N. immutabilis*, (**m**) *N. macrosperma*, and (**n**) *N. violancea*.

The influence of bioclimatic variables on the distribution of water lilies plays a key role in determining the potential distribution areas. In this study, the bioclimatic variables deferred among the species due to geospatial occurrence and ecoregions, leading to varying contributions. The figure below indicates the contribution of each climatic variable per species (Figure 1). For *N. atrans*, the current distribution was mostly favored by bio3 (73%) and bio14 (17.2%), which differed slightly from the contribution of bioclimatic variables in the LGM and the MH. Bio3 (76%) in the LGM, and bio2 (32%), bio14 (35%), and bio15 (30%) in the MH had the highest contribution, respectively. Its future projection indicated the persistent contribution of the bioclimatic variables with less variation (Figure 1a). The contribution of the bioclimatic variables for *N. elleniae* followed a similar pattern to that of *N. atrans* for both current and the past; however, bio2 contributes the highest in all future projections (Figure 1b). Contrary to the two species, the current distribution for *N. georginae* was highly contributed by three bioclimatic variables, bio15 (43.4%), bio3 (34.8%), and bio1 (17.9%). Its past projection was highly contributed by bio15 (60.4% for the LGM and 54.1% for the MH) and the future by bio15 (53.2% for RCP 4.5 (2070)) and bio3 (75.3% for RCP 4.5 (2050), 73%, and 63.1% for RCP 8.5 in both scenarios), respectively (Figure 1c). The current distribution for *N. hastifolia* was highly contributed by bio3 (57.6%), similarly to the LGM (37.5%), where bio15 (35.9%) also contributed highly. Its future distribution was highly favored by bio1, bio2, bio3, and bio15, while bio14 contributed the least in all projected scenarios (Figure 1d). In all projection scenarios for *N. jacobsii*, bio14 contributed the highest, ranging from 82.7% to 99.2%, while bio2 and bio3 contributed the least (Figure 1e). The distribution of *N. pubescens* was largely contributed by bio13 in all projection scenarios (ranging from 50.5 to 70.7%), followed by bio2, which contributed poorly in the LGM (4.7%) and the least bio3, contributing highest in the LGM (25.8%) (Figure 1f). Among the six bioclimatic variables selected for *N. ondinea*, bio13 contributed the highest for the current projection (62.6%), bio13 (41.7%, 61.4%), and bio15 (27.5%, 25.2%) for LGM and MH. The future projection favored bio2 and bio13 for all scenarios, with the least contributing variables being bio1 and bio14 in all scenarios (Figure 1g). Mainly, bio2 contributed to the distribution of *N. alba* in the current and future scenarios, while in the LGM, bio10 (51.2%) contributed the highest. Its distribution was limited by the contribution of bio3 and bio13 in most of the scenarios (Figure 1h). Three bioclimatic variables selected for *N. nouchali* indicate greater contribution to its distribution in all scenarios, especially for bio2 and bio15 (Figure 1i). *N. carpentariae*, as among the widely distributed species, is highly contributed by bio15, ranging greater than 92% in all projections but constrained by both bio2 and bio14, respectively (Figure 1j). Ironically, *N. gigantea* was favored by temperature variables in all scenarios of projection, which contributed relatively higher than in other species (Figure 1k). Like *N. gigantea*, *N. immutabilis* had three climatic variables selected to favor its distribution. In all projections, bio2 and bio15 contributed more than bio14 (Figure 1l). *N. macrosperma* with similar climatic variables indicate a higher preference for bio13 and bio14, unlike *N. jacobsii*, where bio13 was a limiting factor. However, bio2 remains a less contributing factor (Figure 1m). *N. nouchali* had similar variables to *N. gigantea*, but preference in climatic variables was different here. In this case, *N. nouchali* distribution was highly contributed by bio2 and bio15 while limited by bio3 in all projection scenarios. Lastly, *N. violancea* shared common variables with *N. atrans* and *N. elleniae*. However, its contribution was highly influenced by bio15 in all scenarios, followed by bio2, different from *N. atrans* and *N. elleniae* (Figure 1n).

### 2.3. The Current Distribution

**Figure 2:**
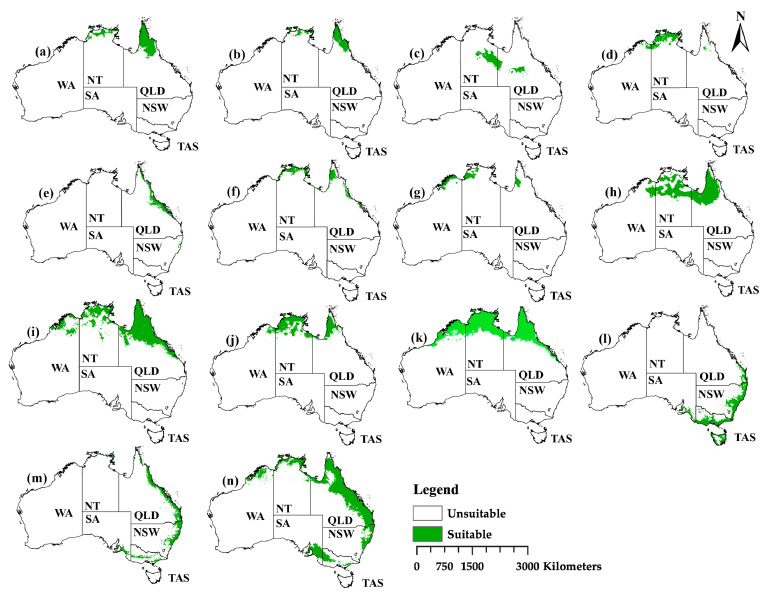
Estimates of the relative contributions of the environmental variables using MaxEnt models, fitted to the current, past and future projections for *Nymphaea* species in Australia. (**a**) *N. atrans*, (**b**) *N. elleniae*, (**c**) *N. georginae*, (**d**) *N. hastifolia*, (**e**) *N. jacobsii*, (**f**) *N. pubescens*, (**g**) *N. ondinea*, (**h**) *N. carpentariae*, (**i**) *N. immutabilis*, (**j**) *N. macrosperma*, (**k**) *N. violancea*, (**l**) *N. alba*, (**m**) *N. nouchali*, and (**n**) *N. gigantea*.

The projected current models indicated that the Northern Territory (NT), Queensland (QLD), New South Wales (NSW), South of Australia (SA), and Tasmania (TAS) favor habitat suitability for *Nymphaea* species in Australia, while Western Australia (WA) was less favorable for the majority of the species. In addition, the projection indicated suitable habitats along the coastline with minimal projection deep inside the country. The majority of the species were projected north of the continent compared to the east and southeast (Figure 2) and with variable habitat suitability (Table 2). Species distributed north of the continent were: (a) *Nymphaea atrans*, (b) *N. elleniae*, (c) *N. georginae*, (d) *N. hastifolia*, (e) *N. jacobsii*, (f) *N. pubescens*, (g) *N. ondinea*, (h) *N. carpentariae*, (i) *N. immutabilis*, (j) *N. macrosperma*, and (k) *N. violancea* while east and southeast were projected for (l) *N. alba*, (m) *N. nouchali*, and (n) *N. gigantea* respectively. Greater habitat suitability was projected for *N. gigantea*, *N. violancea*, *N. immutabilis*, and *N. carpentariae*, while the least for *N. ondinea* (Table 2).

### 2.4. The Projection Changes for the Past and Future Distribution

**Figure 3:**
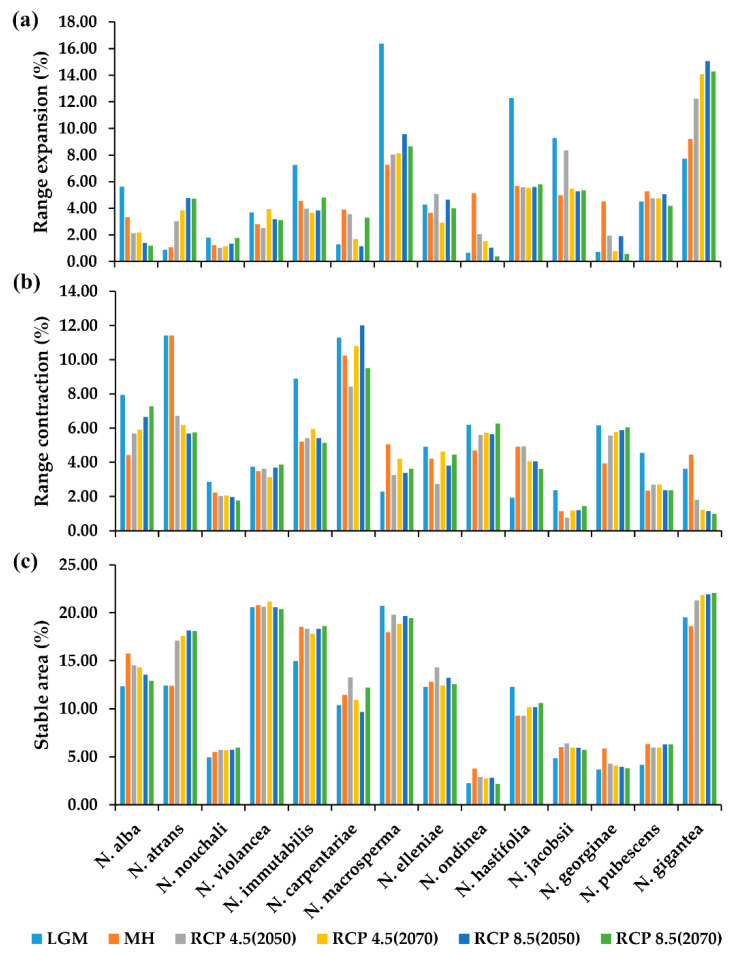
Change in habitat suitability for the past and future projections of water lilies in Australia: (**a**) range expansions, (**b**) range contractions, and (**c**) stable area.

The changes in suitable habitat range for the *Nymphaea* species varied from species to species (Figure 3; Table S2). High range expansions were projected for *N. gigantea*, *N. jacobsii*, *N. macrosperma*, *N. pubescens*, and *N. hastifolia* (Figure 3a) compared to *N. alba*, *N. atrans*, *N. carpentariae*, *N. elleniae*, *N. georginae*, *N. nouchali*, *N. ondinea*, and *N. violancea*, which indicated a greater reduction of suitable habitat ranges in all projection scenarios than expansions (Figure 3b). *N. immutabilis* indicated a relatively stable habitat, although contraction was higher in the MH range. A persistent stable habitat environment was projected in all scenarios among the species. High habitat suitability was projected for *N. violancea*, *N. immutabilis*, *N. macrosperma*, and *N. gigantea*, while the lowest was for *N. ondinea* (Figure 3c).

The expansion ranges for *N. alba* are projected to be greater in the LGM than the MH compared to the future scenarios of RCPs 4.5 and 8.5 in all scenarios, especially in the South of Australia and New South Wales. The island of Tasmania indicated stable ranges and slight expansion ranges in future projections, especially RCP 4.5 and RCP 8.5 of 2070 (Figure S1). The projection of *N. nouchali* indicated expansion ranges in the LGM compared to the MH where expansion ranges were only reflected south of Australia. Therefore, the suitable expansion ranges are projected to contract in all future RCPs and scenarios (Figure S2). The suitable ranges for *N. violancea* were simulated to decrease in the north of Western Australia compared to the MH and expand in the MH in the Northern Territory compared to the LGM. Although the contraction ranges were persistent through the future projection scenarios, greater expansions are noticed in Queensland in RCP 4.5 (2070), although they decline in RCPs 8.5 (Figure S3). The projection of suitable habitat ranges for *N. immutabilis* indicated a loss of stable habitat in the Northern Territory in the LGM compared to all other scenarios, which indicated a gain of stable and expansion ranges. Expansion ranges also reduced north Western Australia in the MH, RCPs 4.5 and RCPs 8.5 compared to the LGM, while the Queensland region maintained stable habitat ranges (Figure S4). The potential habitat ranges for *N. atrans* were observed to contract in the Northern Territory and parts of Queensland in the LGM and MH. The habitat ranges were projected to have a persistent contraction in the future, especially in the Northern Territory, but slight expansions in Queensland were observed in all RCPs and scenarios (Figure S5). *Nymphaea carpentariae* distributed in the same ecoregion as *N. immutabilis* indicate contraction of habitat ranges in parts of the Northern region in the LGM, RCP 4.5 (2070) and RCPs 8.5, while in the MH, RCP 4.5 (2050) indicates expansion. North of Western Australia indicates loss of all suitable habitats in RCP 4.5 (2070) and RCP 8.5 (2050), while parts of Queensland maintain stable habitat range and expansions, especially RCPs 4.5 (2050) and RCPs 8.5 (2070) (Figure S6). Like *N. carpentariae*, the Northern Territory for *N. elleniae* indicated the loss of suitable habitat ranges in the LGM, MH, and all future RCPs. Stable habitats were projected in Queensland and slight expansions (Figure S7). Contrasting *N. carpentariae* and *N. elleniae*, *N. macrosperma* indicated greater expansion in the LGM than the MH and future projection scenarios, especially in the Northern Territory, while stable habitats and expansion were indicated more in Queensland in all projection scenarios (Figure S8). The distribution of *N. ondinea* indicated its vulnerability to habitat loss in all scenarios, especially the LGM and the RCPs 8.5 scenarios. Expansion ranges were noted in the Northern Territory, and Queensland in the MH projected to reduce in both RCPs of 4.5 projection scenarios. Unlike the majority of the projected water lily species, stable habitats were projected north of Western Australia (Figure S9). The distribution of *N. hastifolia* was projected to have greater suitability north of Western Australia and the Northern Territory. These areas mark great expansion areas for *N. hastifolia*, especially for the LGM. However, the suitable habitat range contraction remains high in parts of the Northern Territory and Queensland, such as in the MH and RCPs 4.5 and 8.5 scenarios (Figure S10). The projection of *N. jacobsii* in Queensland indicated greater expansion than contraction ranges in all scenarios and slight contractions northeast of the region (Figure S11). *Nymphaea georginae* was distributed in central Australia, where other water lily species were minimally projected. Its projected habitat ranges increased in the MH compared to the LGM. The future projections indicated increased contraction in RCPs 4.5 (2070) and 8.5 (2070) compared to RCPs 4.5 (2050) and 8.5 (2050) (Figure S12). The distribution of *N. pubescens* had a similar extent to *N. violancea* but a limited geographical area. Its habitat suitability indicated that parts of the Northern Territory in the MH experienced greater expansion compared to the LGM, while contraction was high in Queensland. The future projections indicated more expansions northeast of Queensland in RCP 4.5 (2070), which slightly reduced in RCPs 8.5 scenarios (Figure S13). *Nymphaea gigantea* was projected as the species with the most suitable habitat among the *Nymphaea* species. Its distribution was projected in all regions. The LGM parts of South Australia and some parts in the north experience contraction of suitable habitats, which expand in the MH while the eastern parts of the country experience contraction. In the future projection, expansions were regained east of the continent while increasing further in RCP 4.5 (2070) and RCPs 8.5 in all scenarios (Figure S14). Most water lily species in Australia indicated suitable habitats in the Northern Territory, Queensland, and New South Wales, while fewer had suitable habitats in Western Australia and South Australia. The projections of *N. atrans*, *N. elleniae*, *N. ondinea*, and *N. carpentariae* indicated contraction of suitable habitats, especially in the Northern Territory, compared to other projected species.

## 3. Discussion

Ecological niche models have been used recently in evaluating the effect of climate change on species distribution [32]. In this case, MaxEnt was preferred for its ability to use presence-only data and its ability to provide the user with an option for setting omission errors. The models indicated that temperature and precipitation variables, particularly bio2, bio3, bio13, bio14, and bio15, were the most influencing factors in the species distribution. At the regional level, the climatic variables are known to vary considerably [33], which indicated the different preferences and contributions of the climatic variable differently among the water lily species.

Most water lily species were distributed in the north, east, and southeast regions of Australia (Figure 2). The northern region comprises diverse freshwater ecosystems ranging from complex flood plain rivers to Kimberly bedrock-controlled rivers and isolated upland streams [34]. The flood plains in the area account for 30% of the total area prone to sea-level rise. A clear example is Kakadu wetlands, approximated to be between 0.2 and 1.2 m [35]. The approximated sea-level rise is 0.3 m by 2030 [34] and 0.82 m by 2100 [35], which is likely to submerge parts of the floodplains affecting water lily species distribution. This may also increase the chances of saltwater intrusion on the wetlands, leading to floristic change, as earlier observed with mangroves [35], and possibly affecting the majority of aquatic species in the north of the continent, leading to declining habitat suitability, as projected for most *Nymphaea* species in the region. The approximated range temperature for the region is 30 °C to 33 °C, which can exceed 37 °C in dry seasons [36,37]. The projected warming and temperature rise cause the rivers and streams to cease to flow, increasing water temperature that may exceed physiological tolerance for the aquatic species [34]. This can also affect species’ suitable habitats, leading to shifting, contraction, or expansion in their habitats as they respond differently to climate change. For example, in all projection scenarios, *N. atrans*, *N. carpentariae*, *N. elleniae*, and *N. ondinea* were projected to experience a reduction of habitat suitability in the region.

Parts of southeast Australia provide a suitable habitat for the water lily species from the diversity of freshwater rivers and streams. The region experienced a variable climate, with precipitation being at the peak during winter and spring, followed by drought [38], likely affecting the lowland freshwater ecosystem. This can indicate why most parts of New South Wales experience contraction, such as in *N. nouchali* and *N. alba*. Furthermore, climate change was projected to cause a significant decline in precipitation and an increase in temperature in the region, causing reduced runoff, which replenishes water holes, thus limiting ground recharge. It has been further projected to increase evapotranspiration leading to increased drought [34]. The effects of climate change along with the anthropogenic interventions add immense pressure to the survival of aquatic species such as water lilies.

The majority of the projected water lily species apart from *N. georginae* indicate increased contraction of suitable climatic ranges, especially in southeastern Australia. The habitat contraction is associated with the declining average annual rainfall in the region [39], which is causing reduced runoff, less ground recharge, drying of small water streams, and increasing drought conditions, which increase evaporation and dying of the isolated water pools [25,38]. In the region, the average warming was projected to reach 2 °C higher than in 1990–2000 [33], while the area favors *N. gigantea* expansion. The contribution of bioclimatic variables selected for *N. gigantea* contributes favorably to its suitable habitat, explaining why the species will continue to flourish in the region. *Nymphaea gigantea* was also projected to expand its habitat ranges, especially in the Northern Territory, while most *Nymphaea* species are declining in suitable habitat ranges, indicating that climate warming causes species abundance and colonization of new ecosystems.

Tasmania indicated the future projection of stable and suitable habitat for *N. alba*, probably due to the slight warming compared to temperatures over the land [40]. However, as climate change progresses, there is a likelihood of the decline of stable habitats. This is in comparison with the future projection of the other projected areas for *N. alba*, which have already demonstrated a decline in suitable habitats. Moreover, freshwaters in Tasmania were projected to experience reduced runoff (15% and 35%) that will influence the ecosystems significantly by 2100 [41].

Suppiah et al. (2007) [39] indicated that annual air temperature change at medium-emission scenarios (2050) in regions of Western Australia is approximated to reach 2.5 °C while the rest of the continent is approximately 2 °C. In the 2070 medium-emission scenario, temperatures were projected to rise to approximately 3–4 °C, extending to most regions of Australia and across all the states. In the high-emission scenarios of 2050, most parts of the continent experience a temperature rise of about 2.5 °C, which in 2070 was projected to rise to above 3 °C. The temperature change was also noticed with the change of the water lily suitable habitats in Australia. For example, the rise in temperature (bio2) was projected to reduce suitable habitats for *N. alba* in the MH and the future scenarios compared to the LGM. This might have affected the decline in suitable habitats east of Australia in Queensland, North–South Wales, and in the future in South Australia. A slight increase in bio3 in the LGM also indicated suitable habitat range contraction in parts of South Australia and Tasmania. The distribution of *N. georginae* is highly influenced by bio15 in the MH, which indicates that the seasonal precipitation played a key role in the sustainability of species habitats. However, the area is projected to have a temperature rise of greater than 2 °C from a medium-emission scenario [33,39].

Contrasting the majority of water lily species, *N. georginae* was projected “interior” of the Australian continent, meaning further from the coastline an area characterized by arid and semiarid environments. This area persists from water holes replenished in summer rains when rains and floods occur [39]. However, habitat suitability for *N. georginae* reduced in the future with temperature rise and precipitation reduction, especially in high-emission scenarios approximated to have a temperature rise of greater than 2.5 °C and precipitation reduction of 20% [39]. The habitat suitability analysis revealed the influence of bioclimatic variables on the distribution of *Nymphaea* species, which will be important for future conservation and management initiatives [42], particularly for species with declining habitat suitability.

Lastly, we used occurrence points acquired from GBIF and other online sources that can sometimes be the source of inadequacies in species distribution modeling. This is because they are prone to error and, in many instances, are biased in sampling. However, the points were carefully filtered. In addition, the future projection was carried out only in one general circulation model, which can be a source of projection uncertainty that should be deliberated in the future study.

## 4. Materials and Methods

### 4.1. Distribution Data

**Figure 4:**
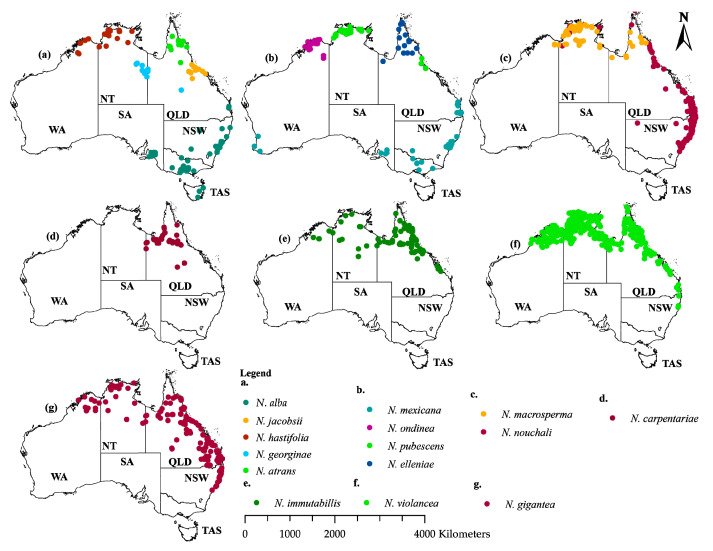
Species occurrence points were used in the habitat suitability modeling of the fourteen *Nymphaea* species in Australia. The abbreviations represent Western Australia (WA), North Territory (NT), South Australia (SA), Queensland (QLD), New South Wales (NSW), and Tasmania (TAS).

The spatial geographical occurrence points for the species were obtained from the Global Biodiversity Information Facility (GBIF), the Australia living Atlas, and existing literature [40]. All points were then cleaned for duplicates, un-georeferenced coordinates, coordinates before 1950, points within zero degrees, and outliers in R environment [43]. Species coordinates presumed cultivated (categorized as cultivated) were also eliminated, although they were less in number and would not significantly change the results. To eliminate geographic autocorrelation, the occurrence points were spatially rarified to a grind of 5 × 5 km cells to ensure a single occurrence point per cell using the spThin package in R [44] and to complement the climatic variables. This step also helps reduce sampling biases, mostly due to sampling in easily accessible areas such as along the roads, near cities, institutions, and less risky areas [15]. For this study, we considered occurrence points above ten. After the process of data filtering, 513 points were retained for *N. violancea*, *N. nouchali* (207), *N. gigantea* (171), *N. immutabilis* (103), *N. macrosperma* (79), *N. pubescens* (53), *N. alba* (51), *N. carpentariae* (30), *N. ondinea* (33), *N. hastifolia* (25), *N. elleniae*, (21), *N. atrans* (14), *N. georginae* (13), and *N. jacobsii* (13) (Figure 4; Table S3).

### 4.2. Climatic Data

Nineteen bioclimatic variables at a spatial resolution of 2.5 arcsec (5 km spatial resolution) were acquired from the WorldClim (v 1.4) database (www.worldclim.org, accessed on 30 June 2022 [45] for the current distribution). The past was represented by the Last Glacial Maxima (LGM) and the Mid-Holocene (MH), while future habitat suitability was assessed using representative concentration pathways (RCPs) 4.5 and 8.5, representing medium-low and high carbon emission scenarios [46]. RCP 4.5 was chosen to represent a moderate radiative forcing level (i.e., leading to 4.5 W/m^2^ greenhouse gas levels or ~650 ppm CO_2_ eq. by the year 2100), while RCP 8.5 was selected as a high radiative forcing level (i.e., 8.5 W/m^2^ or ~1370 ppm CO_2_ eq. by the year 2100) [46].

The variables are derived from monthly temperature and precipitation from 1950 to 2000 [45] to represent annual trends and seasonality from the Community Climate System Model (CCSM4) from the Coupled Model Intercomparison Project (CMIP5 [47]). The bioclimatic variables (Table S4) were then masked per species occurrence points (M region of the BAM diagram [48] using Australian freshwater ecoregions [49]. The enacted climatic variables were then tested for multicollinearity using the Pearson correlation coefficient in VIF (|r| > 0.70) using a function implemented in the usdm R package [50] to avoid “overfitting” in the models. The non-independent variables were removed from further analysis, while the selected variables among the species were: annual mean temperature (bio1), mean diurnal range (bio2), isothermality (bio3), the maximum temperature of the warmest month (bio5), mean temperature of warmest quarter (bio10), precipitation of wettest month (bio13), precipitation of driest month (bio14), and precipitation seasonality (bio15).

### 4.3. Model Building and Evaluation

In this study, we used MaxEnt v3.4.1 [51] to assess habitat suitability for the water lilies. The method has been widely and effectively used as a reliable tool for ecological niche modeling, mostly because it utilizes environmental and species presence data to extract meaningful information based on the species’ spatial locations to simulate possible habitat suitability areas with an option of setting for omission errors [51]. For increased predictive ability of the model, regularization multiplier (rm) and feature classes (fc) were selected using the R package ENMeval using “checkboard2” from five different fcs, (i) L, (ii) LQ, (iii) LQH, (iv) LQHP, and (v) LQHPT (where L = linear, Q = quadratic; H = hinge; P = product, and T = threshold), at an rm of 0.5 to 4 at increment of 0.5 [52]. Other adjusted settings included maximum background points of 10,000, maximum interactions of 5000, and a 10-5 convergence threshold. We avoided the default setting because MaxEnt models are proposed to get overfitted [53]. The models were calibrated using 75% of occurrence points as training data and the remainder as test data at replicates of 10 and cross-validation method. We employed a leave-one-out approach for samples less than 25 [54]. All other settings were kept at default. Maximum training sensitivity plus specificity (MTSS) was used to produce continuous maps at probability values of presence (1) and absence (0) areas. Lastly, the jackknife test was used to assess the contribution and the importance of the variables to the geographical distribution of the species [55].

The model was evaluated using the area under the curve (AUC) [56]. The AUC measures the overall model accuracy from 0 to 1; the closer it is to 1, the more discriminatory the model is between the absence and presence of the species. The species’ suitable habitat areas and changes were evaluated by converting the continuous maps into binary maps of presence–absence (0 or 1) to represent suitable or unsuitable areas using MTSS, preferred for maintaining zero-omission error in the training dataset [56].

### 4.4. Distribution of Habitat Suitability

The potential suitable habitat gains and loss were calculated using Spatial Analyst tools in ArcGis 10.8 [32] by reclassifying the past, current, and future distribution maps (raster’s) into binary maps representing presence (1) and absence (0) using MTSS threshold values obtained from the MaxEnt model (Table 4).

## 5. Conclusions

Species habitat prediction is important to understand species responses to climate change, especially for species with insufficient data and monitoring. This information helps in conservation planning and species habitat management. As global warming rises, land surface temperature rises as well. As a result, species’ distribution and the suitability of habitats are affected, causing species populations to decline, threatening them with extinction. In addition, aquatic ecosystems become more vulnerable to drying out and overdependence on commercial and domestic uses, which also threatens species’ habitats. In Australia, the distribution of water lily species is specific to certain regions in favor of habitat suitability. Although species are assumed to evolve in adaptation to the habitat environment, habitat suitability loss with minimum expansion is a threat to species populations. An example is the projected habitat loss north of the continent, especially *N. carpentariae*, which should attract the attention of conservation practitioners. Conservation actions are to be taken, bearing in mind that the species are distributed in areas with high population density and economically viable areas. Moreover, a comprehensive statistical approach is required for effective conservation of species, and therefore, from our predictive modeling of the water lily species, more studies focusing on the land-use change, population trends, and survival ability of the species should be incorporated into future studies.

## Figures and Tables

**Table 1:** Climatic variables were retained after eliminating the correlation using variance inflation factors (VIFs).

Species	Bioclimatic Variables							
	bio1	bio2	bio3	bio5	bio10	bio13	bio14	bio15
*N. atrans*	1.650	1.426	1.442	-	-	-	1.975	1.873
*N. carpentariae*	-	1.687	-	-	-	-	2.555	2.048
*N. elleniae*	1.616	1.405	1.442	-	-	-	1.973	1.948
*N. georginae*	1.706	1.505	1.440	-	-	-	2.165	1.831
*N. gigantea*	-	2.029	1.186	2.032	-	-	-	-
*N. hastifolia*	1.839	1.331	1.348	-	-	-	1.818	1.961
*N. immutabilis*	-	1.556	-	-	-	-	2.371	1.947
*N. jacobsii*	-	2.647	-	-	-	2.584	2.272	-
*N. macrosperma*	-	2.140	-	-	-	1.910	1.809	-
*N. ondinea*	2.326	2.297	1.620	-	-	6.540	4.597	6.692
*N. violancea*	-	1.642	1.596	-	-	-	2.564	2.555
*N. nouchali*	-	1.902	1.128	1.859	-	-	-	-
*N. alba*	-	3.861	2.244	-	3.908	3.428	-	4.659
*N. pubescens*	-	2.515	3.739	-		3.514	2.843	-

**Table 2:** Feature classes selected for the modeling of the *Nymphaea* species in Australia.

Species	Features Class	rm Value	Current Habitat Suitability (Km^2^)
*N. alba*	LQ	0.5	564,703.2154
*N. atrans*	LQH	1.5	288,466.6287
*N. carpentariae*	LQHP	1.5	814,252.6658
*N. elleniae*	LQHP	3	206,789.3887
*N. georginae*	LQH	1	192,787.1066
*N. gigantea*	LQHP	0.5	1,303,516.112
*N. hastifolia*	LQHP	1.5	199,145.3796
*N. immutabilis*	LQH	1	948,548.1007
*N. jacobsii*	LQH	2.5	142,273.8545
*N. macrosperma*	LQH	0.5	427,824.8378
*N. nouchali*	LQHP	0.5	453,647.2079
*N. ondinea*	LQH	1	120,521.2861
*N. pubescens*	LQHPT	1	162,148.2411
*N. violancea*	LQHP	0.5	1,137,450.324

**Table 3:** The AUC values for training (75%) and test (25%) data for the *Nymphaea* species distribution suitability in Australia using the maximum training sensitivity plus specificity (MTSS) logistic threshold. In brackets: standard deviation values.

Species	Current	RCP 4.5 (2050)	RCP 4.5 (2070)	RCP 8.5 (2050)	RCP 8.5 (2070)	LGM	MH
*N. alba*	0.876 (0.030)	0.850 (0.026)	0.875 (0.030)	0.887 (0.043)	0.844 (0.049)	0.849 (0.032)	0.897 (0.021)
*N. atrans*	0.852 (0.133)	0.859 (0.063)	0.903 (0.061)	0.870 (0.081)	0.906 (0.069)	0.912 (0.041)	0.876 (0.075)
*N. carpentariae*	0.897 (0.060)	0.907 (0.033)	0.888 (0.054)	0.880 (0.047)	0.918 (0.045)	0.888 (0.043)	0.897 (0.020)
*N. elleniae*	0.858 (0.053)	0.887 (0.053)	0.878 (0.056)	0.899 (0.057)	0.894 (0.051)	0.892 (0.056)	0.873 (0.062)
*N. georginae*	0.933 (0.060)	0.929 (0.071)	0.919 (0.065)	0.952 (0.063)	0.923 (0.094)	0.949 (0.051)	0.929 (0.066)
*N. gigantea*	0.829 (0.017)	0.843 (0.020)	0.849 (0.030)	0.845 (0.027)	0.857 (0.035)	0.864 (0.016)	0.838 (0.016)
*N. hastifolia*	0.850 (0.058)	0.863 (0.069)	0.890 (0.045)	0.871 (0.047)	0.853 (0.071)	0.824 (0.189)	0.876 (0.069)
*N. immutabilis*	0.883 (0.022)	0.906 (0.011)	0.882 (0.023)	0.898 (0.016)	0.893 (0.033)	0.875 (0.019)	0.903 (0.025)
*N. jacobsii*	0.930 (0.049)	0.936 (0.032)	0.938 (0.055)	0.947 (0.031)	0.947 (0.048)	0.912 (0.035)	0.919 (0.035)
*N. macrosperma*	0.899 (0.022)	0.890 (0.021)	0.902 (0.023)	0.907 (0.031)	0.902 (0.020)	0.869 (0.023)	0.908 (0.020)
*N. nouchali*	0.959 (0.005)	0.956 (0.013)	0.956 (0.012)	0.959 (0.011)	0.963 (0.010)	0.945 (0.014)	0.955 (0.012)
*N. ondinea*	0.901 (0.065)	0.913 (0.053)	0.946 (0.047)	0.934 (0.058)	0.950 (0.045)	0.948 (0.040)	0.912 (0.043)
*N. pubescens*	0.962 (0.020)	0.942 (0.019)	0.960 (0.012)	0.948 (0.015)	0.959 (0.006)	0.941 (0.030)	0.929 (0.018)
*N. violancea*	0.896 (0.009)	0.903 (0.009)	0.908 (0.006)	0.901 (0.008)	0.907 (0.006)	0.884 (0.007)	0.900 (0.008)

**Table 4:** Maximum training sensitivity plus specificity (MTSS) Values used in classifying MaxEnt output raster files into suitable and unsuitable habitats.

Species	Current	LGM	MH	RCP 4.5 (2050)	RCP 8.5 (2050)	RCP 4.5 (2070)	RCP 8.5 (2070)
*N. alba*	0.3045	0.4618	0.2835	0.3045	0.3209	0.2957	0.2864
*N. atrans*	0.3649	0.5166	0.4495	0.3649	0.3931	0.3851	0.3282
*N. carpentariae*	0.2657	0.2762	0.3093	0.2657	0.2427	0.2323	0.267
*N. elleniae*	0.427	0.4036	0.3695	0.427	0.3686	0.4071	0.3776
*N. georginae*	0.4203	0.4576	0.3858	0.4203	0.3524	0.3354	0.4671
*N. gigantea*	0.3244	0.2812	0.3083	0.3244	0.2374	0.2307	0.2477
*N. hastifolia*	0.5105	0.3385	0.476	0.5105	0.4554	0.4571	0.41
*N. immutabilis*	0.263	0.2779	0.2242	0.263	0.2589	0.1886	0.2051
*N. jacobsii*	0.5673	0.597	0.5929	0.5673	0.5944	0.6119	0.5631
*N. macrosperma*	0.1733	0.1669	0.1807	0.1733	0.1618	0.1857	0.1686
*N. nouchali*	0.145	0.1523	0.1305	0.145	0.129	0.1268	0.1283
*N. ondinea*	0.4065	0.4328	0.3101	0.4065	0.3521	0.3685	0.4189
*N. pubescens*	0.1814	0.349	0.2098	0.1814	0.2221	0.2264	0.229
*N. violancea*	0.2178	0.2379	0.1992	0.2178	0.1725	0.1666	0.1933

## Data Availability

All data presented in this study are available in the article and in the supplementary materials.

## Supplementary Materials

The following supporting information can be downloaded at: https://www.mdpi.com/article/10.3390/plants11141874/s1, Figure S1–Figure S14: The potential distribution change of 14 *Nymphaea* species for the past and future projection scenarios. (Figure S1) *N. alba*, (Figure S2) *N. nouchali*, (Figure S3) *N. violancea*, (Figure S4) *N. immutabilis*, (Figure S5) *N. atrans*, (Figure S6) *N. carpentariae*, (Figure S7) *N. elleniae*, (Figure S8) *N. macrosperma*, (Figure S9) *N. ondinea*, (Figure S10) *N. hastifolia*, (Figure S11) *N. jacobsii*, (Figure S12) *N. georginae*, (Figure S13) *N. pubescens*, (Figure S14) *N. gigantea*, and Table S1: The AUC values for training (75%) and test (25%) data for the *Nymphaea* species distribution suitability using the maximum training sensitivity plus specificity (MTSS) logistic threshold. The AUC values describe the fitness of the model in predicting the species distribution. Table S2: Distribution change of water lily species (*Nymphaea*) in Australia, obtained by comparing binary changes between the current potential distribution with the past and the future changes. Table S3: The distribution localities for the *Nymphaea* species used for this study. Table S4: The nineteen climatic variables obtained from WorldClim v1.4.
