# Supplementary material for: Ecological Niche Modeling of Water Lily (Nymphaea L.) Species in Australia under Climate Change to Ascertain Habitat Suitability for Conservation Measures
Source: Plants (Basel). 2022 Jul 19;11(14):1874. doi: 10.3390/plants11141874 (PMC9322643; doi:10.3390/plants11141874)
Supplement: Supplementary file 1 [file plants-11-01874-s001.zip › plants-1821868-supplementary.pdf]

Figure S1–Figure S14. The potential distribution change of 14 Nymphaea species for the past and future projection scenarios.

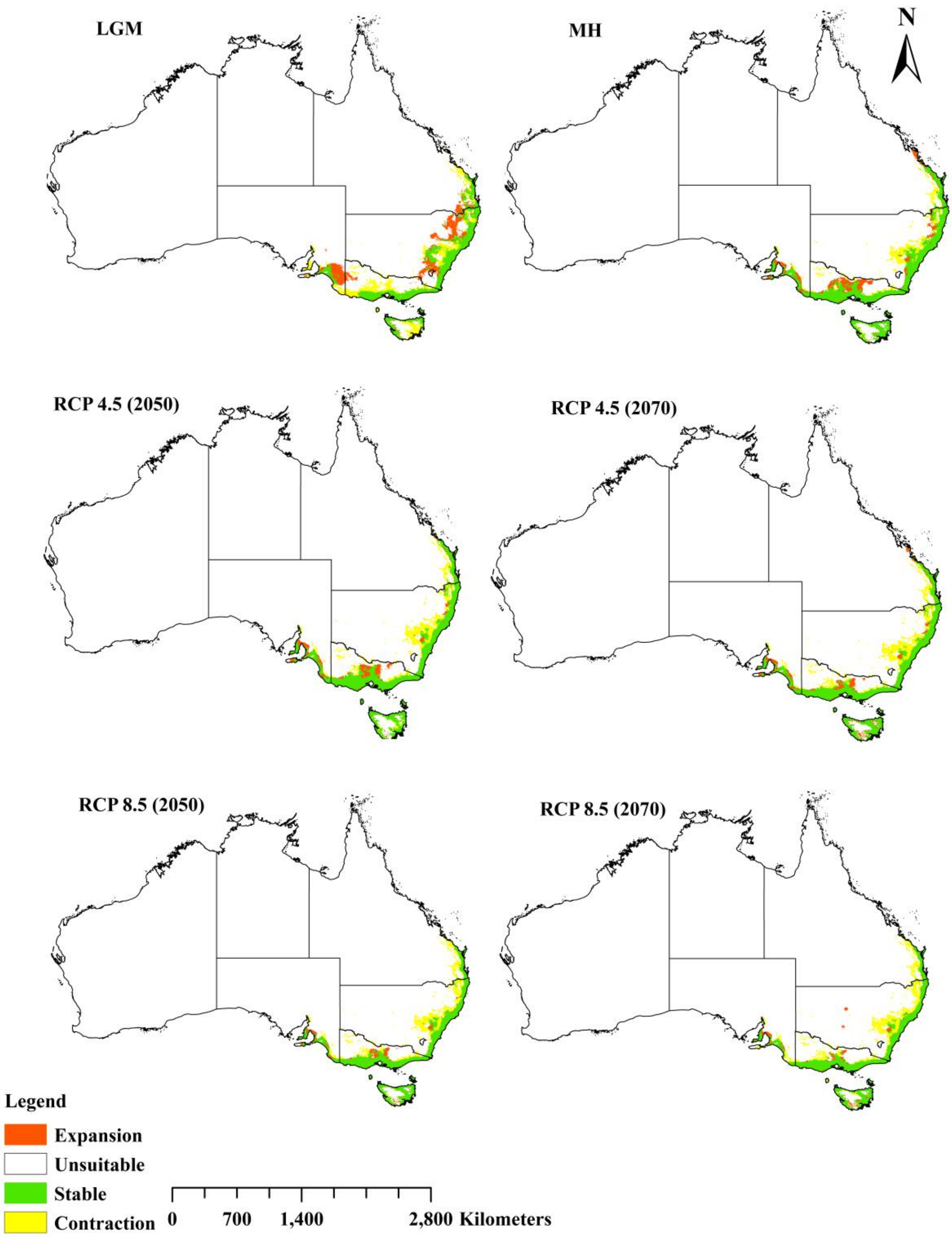

Figure S1. *N. alb*.

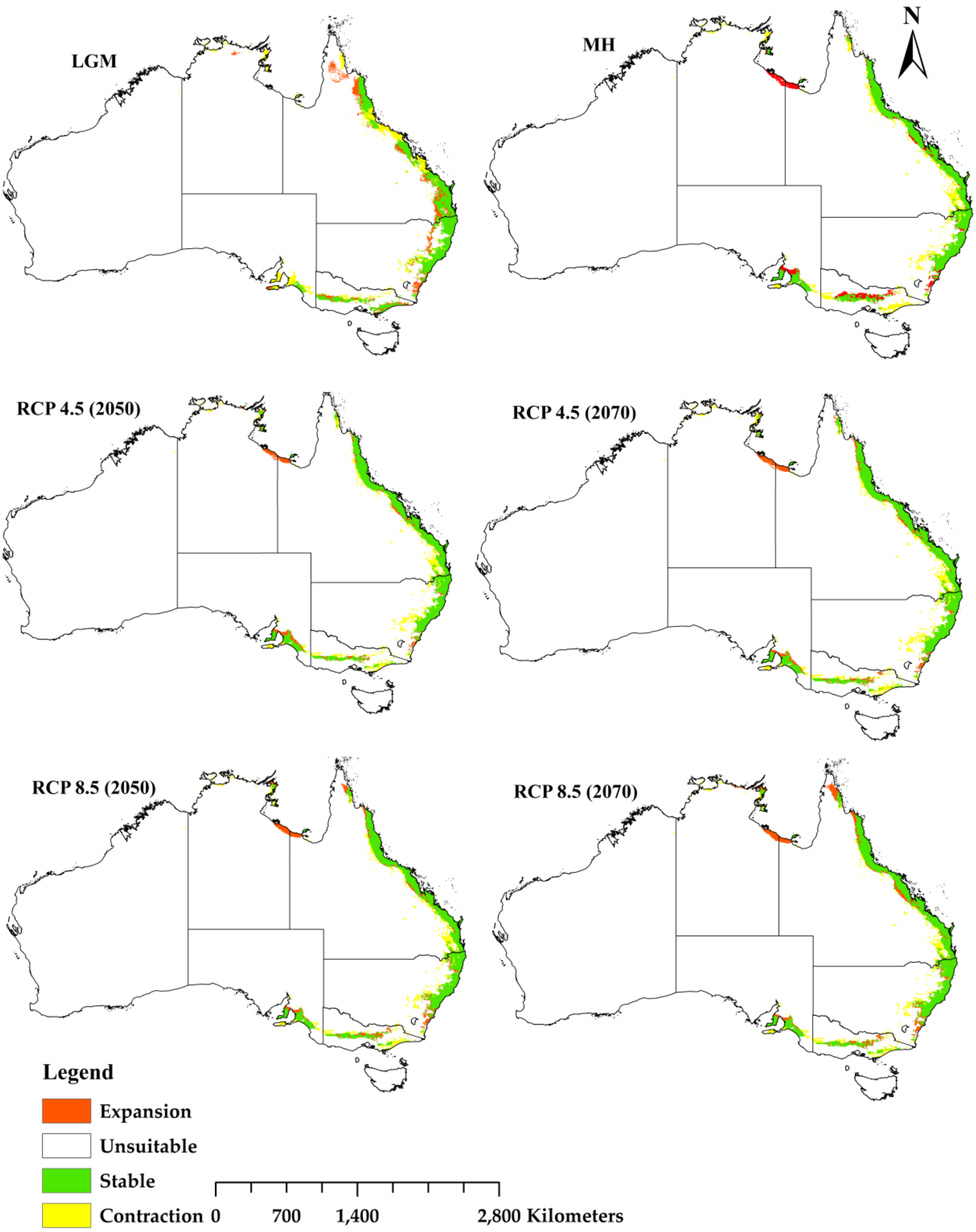

Figure S2. *N. nouchali*.

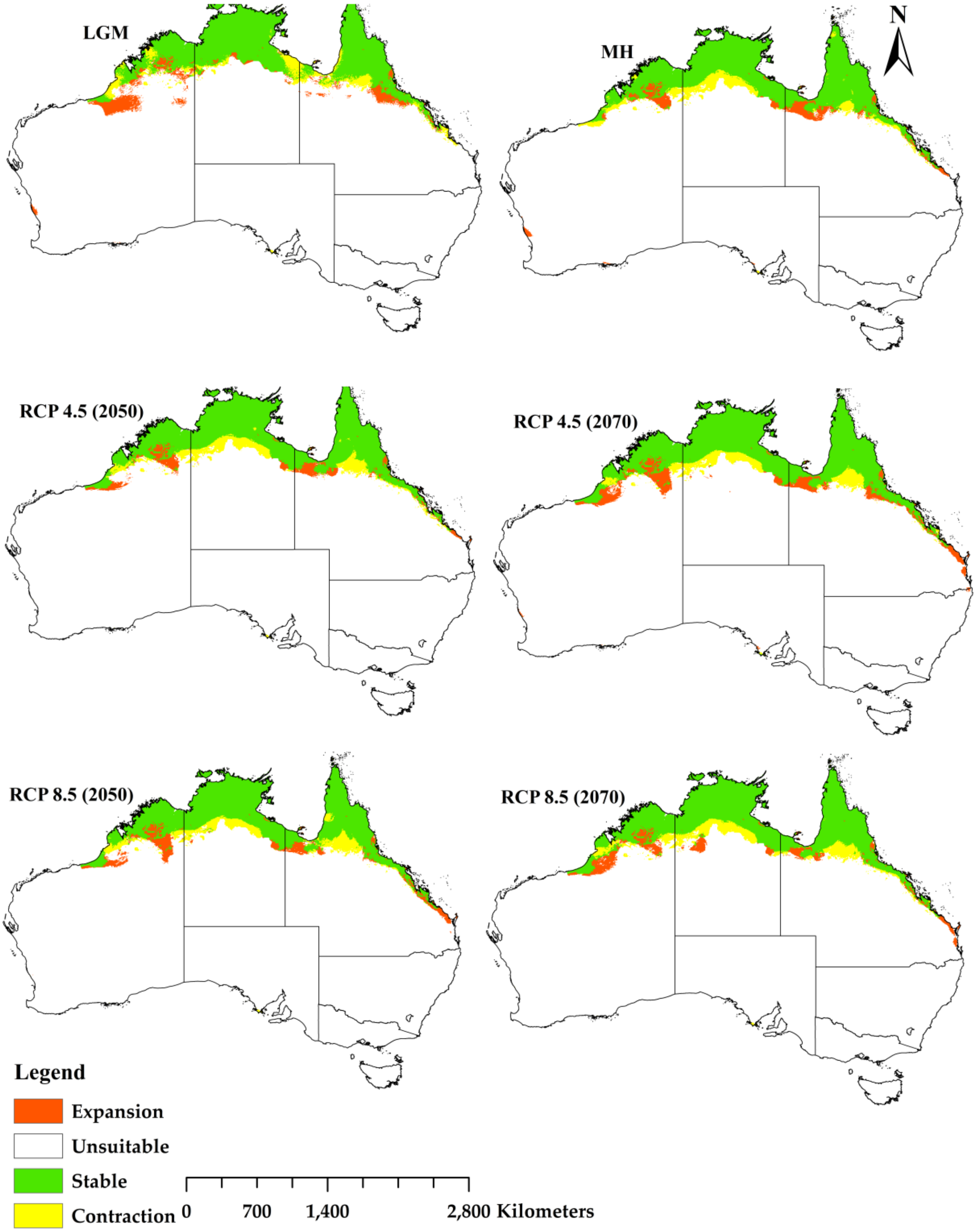

Figure S3. *N. violancea*.

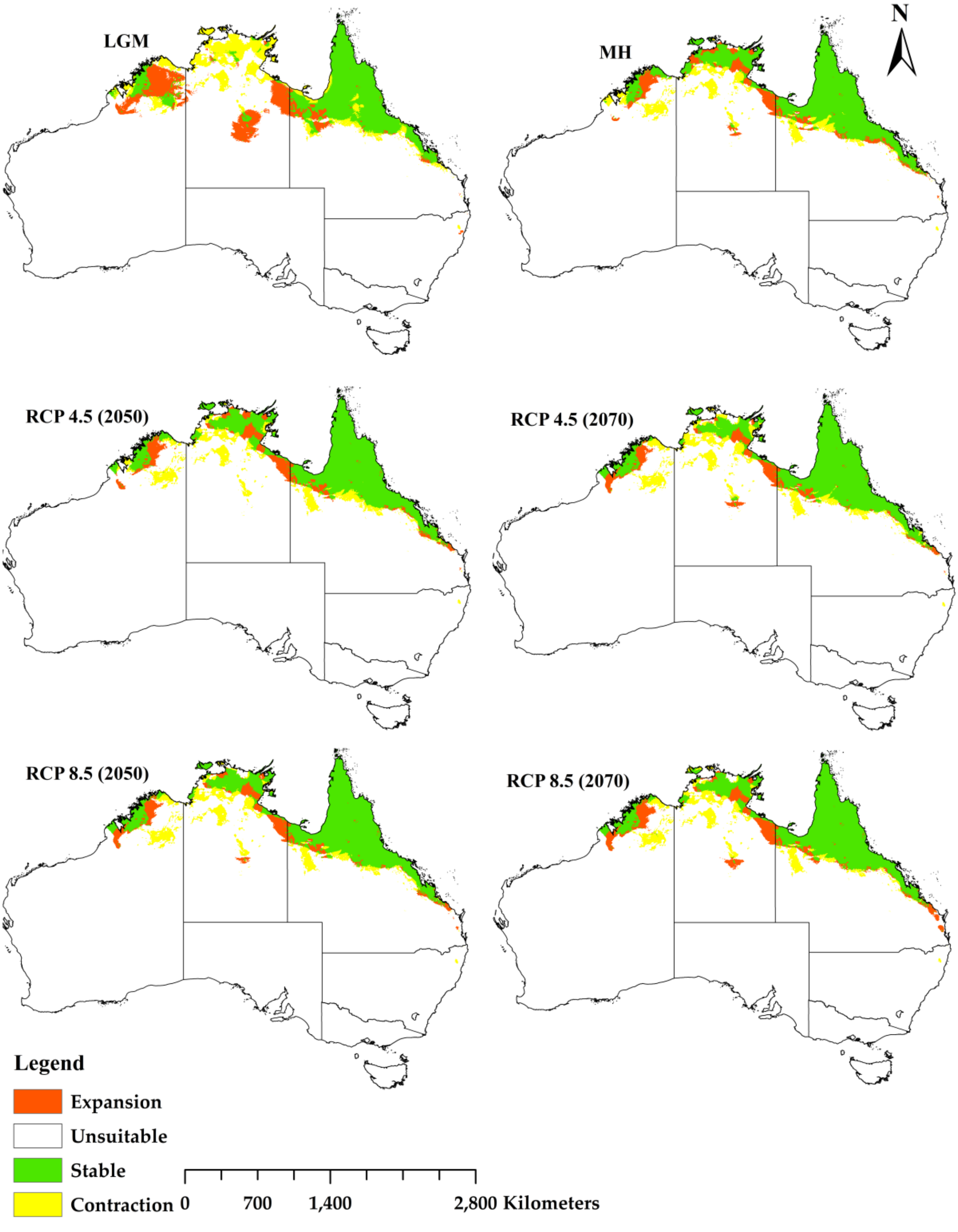

Figure S4. *N. immutabilis*.

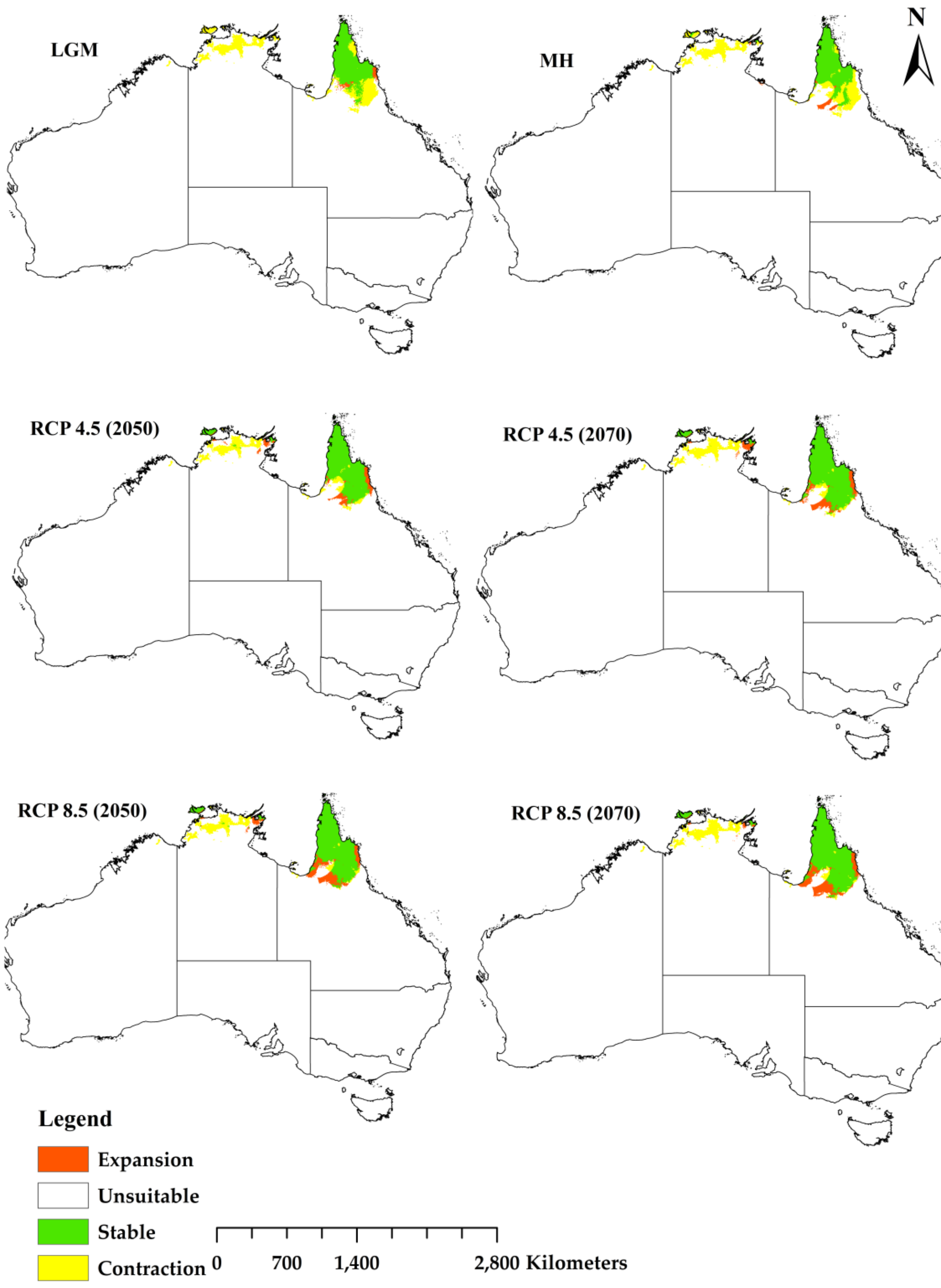

Figure S5. *N. atrans*.

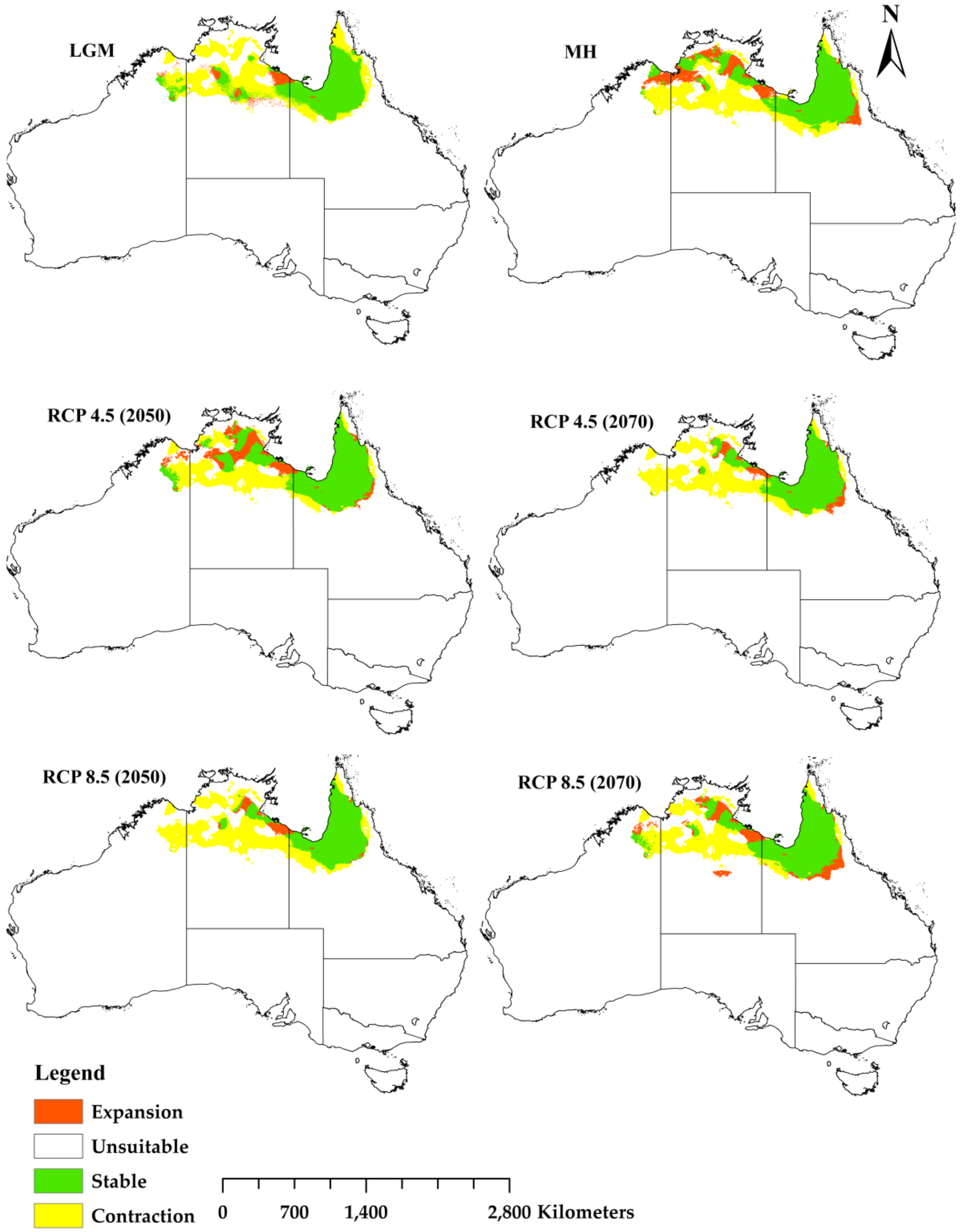

Figure S6. *N. carpentariae*.

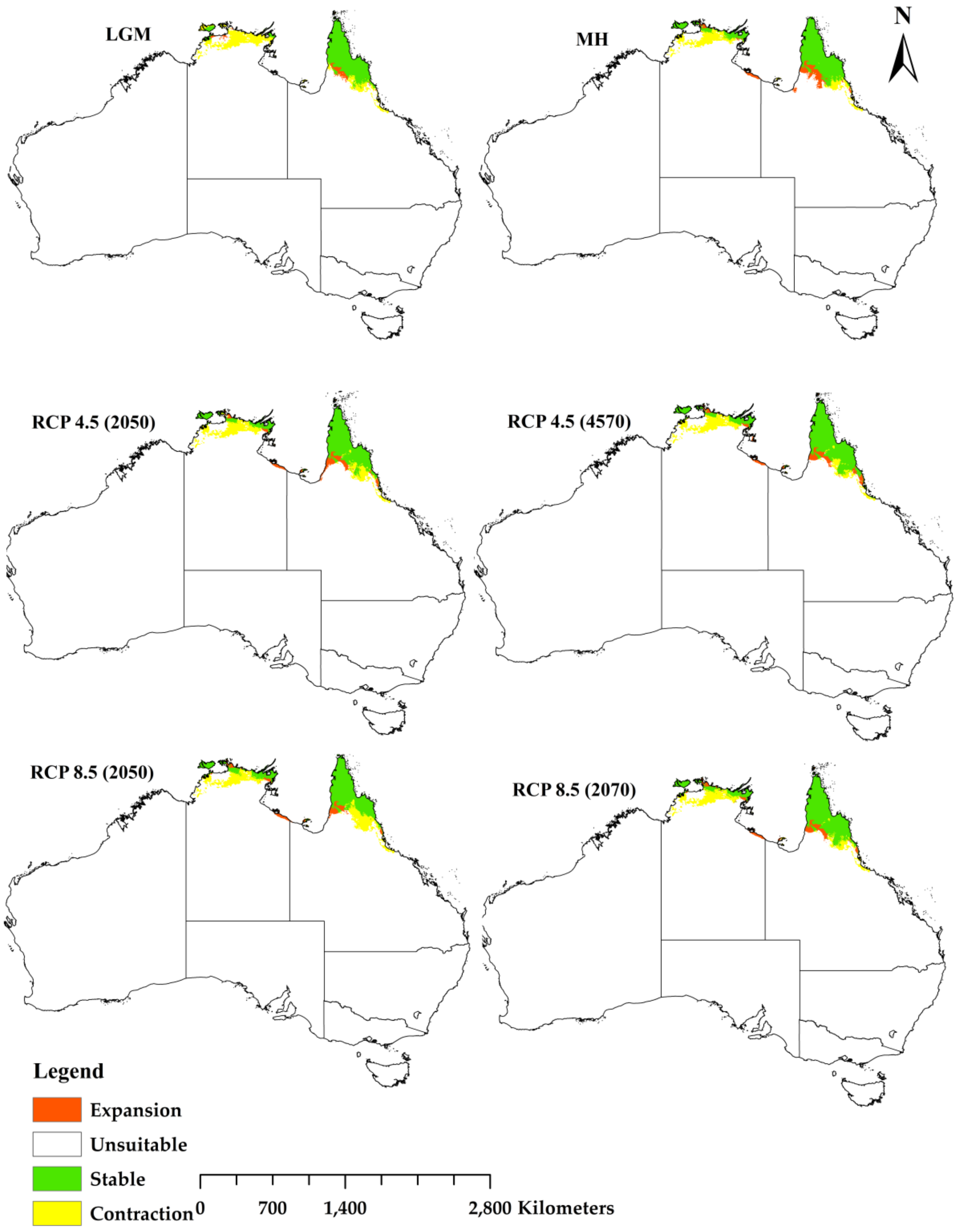

Figure S7. *N. elleniae*.

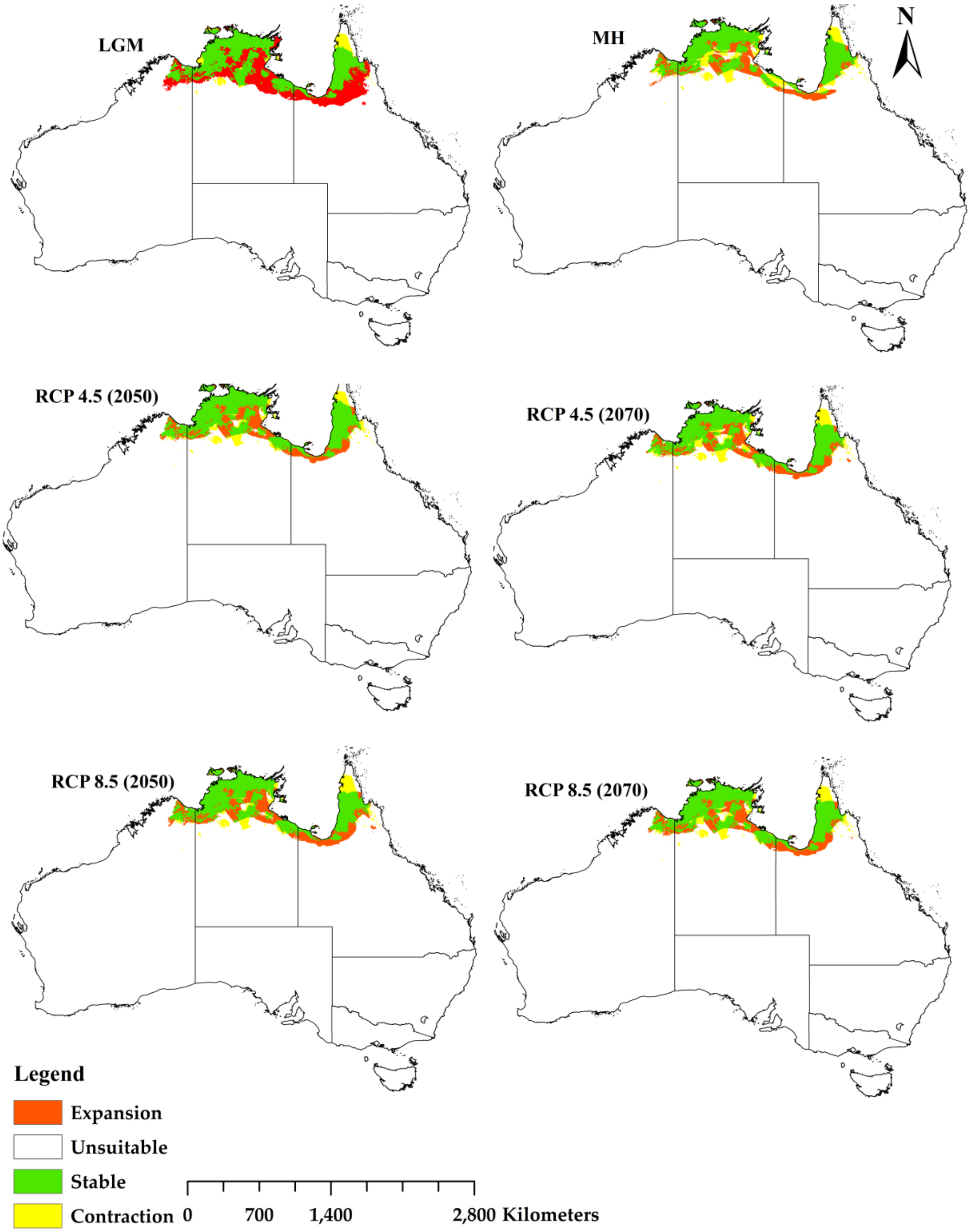

Figure S8. *N. macrosperma*.

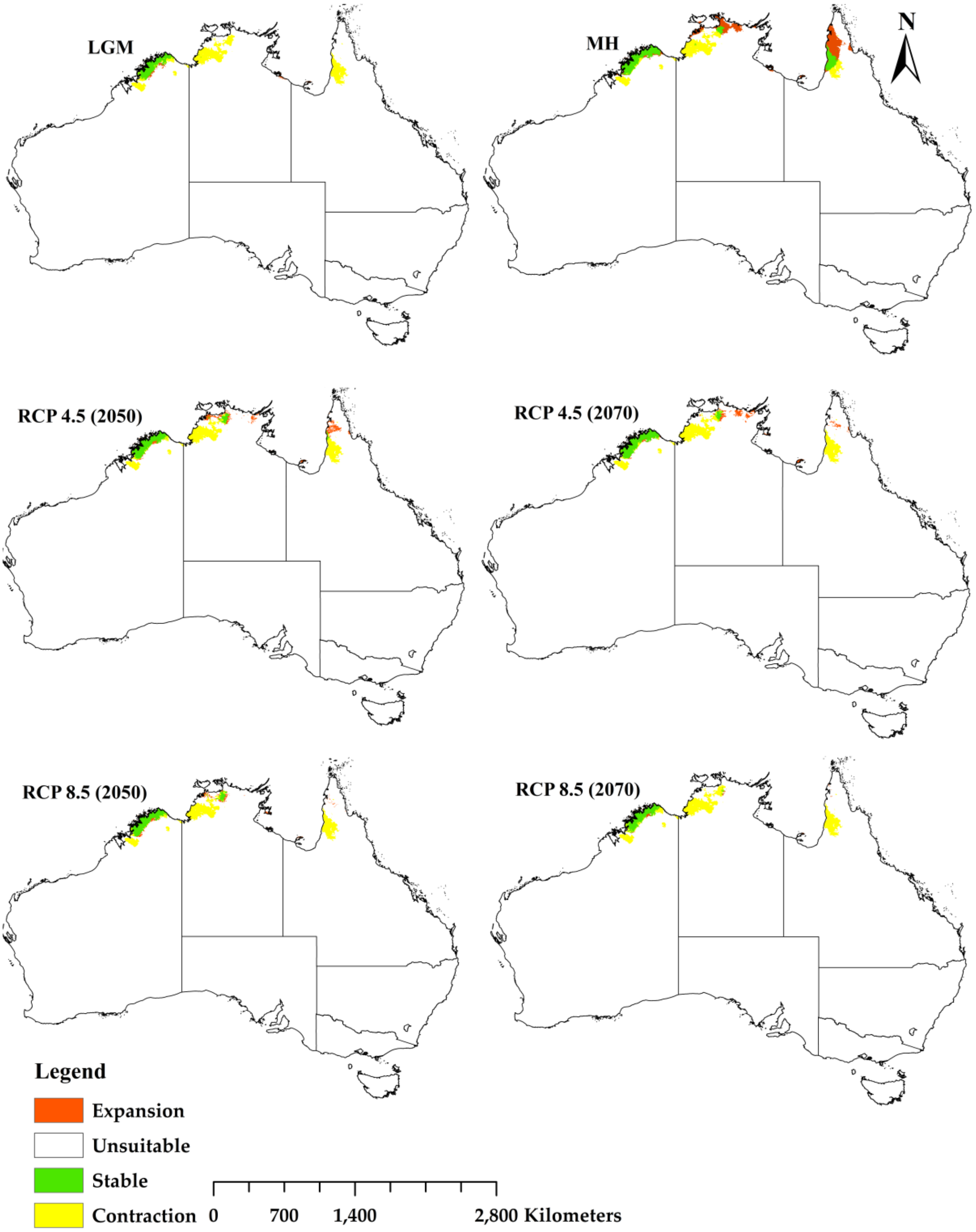

Figure S9. *N. ondinea*.

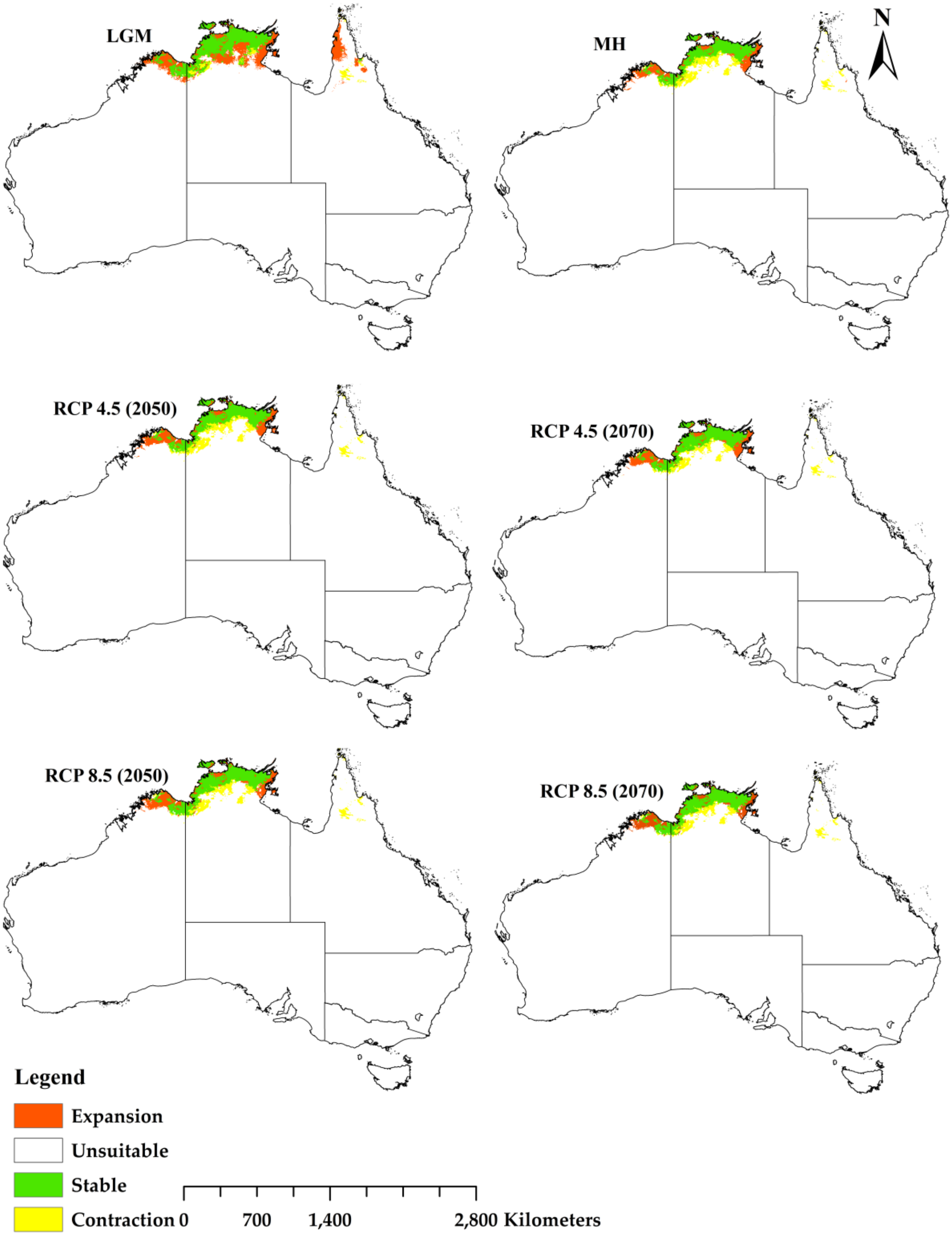

Figure S10. *N. hastifolia*.

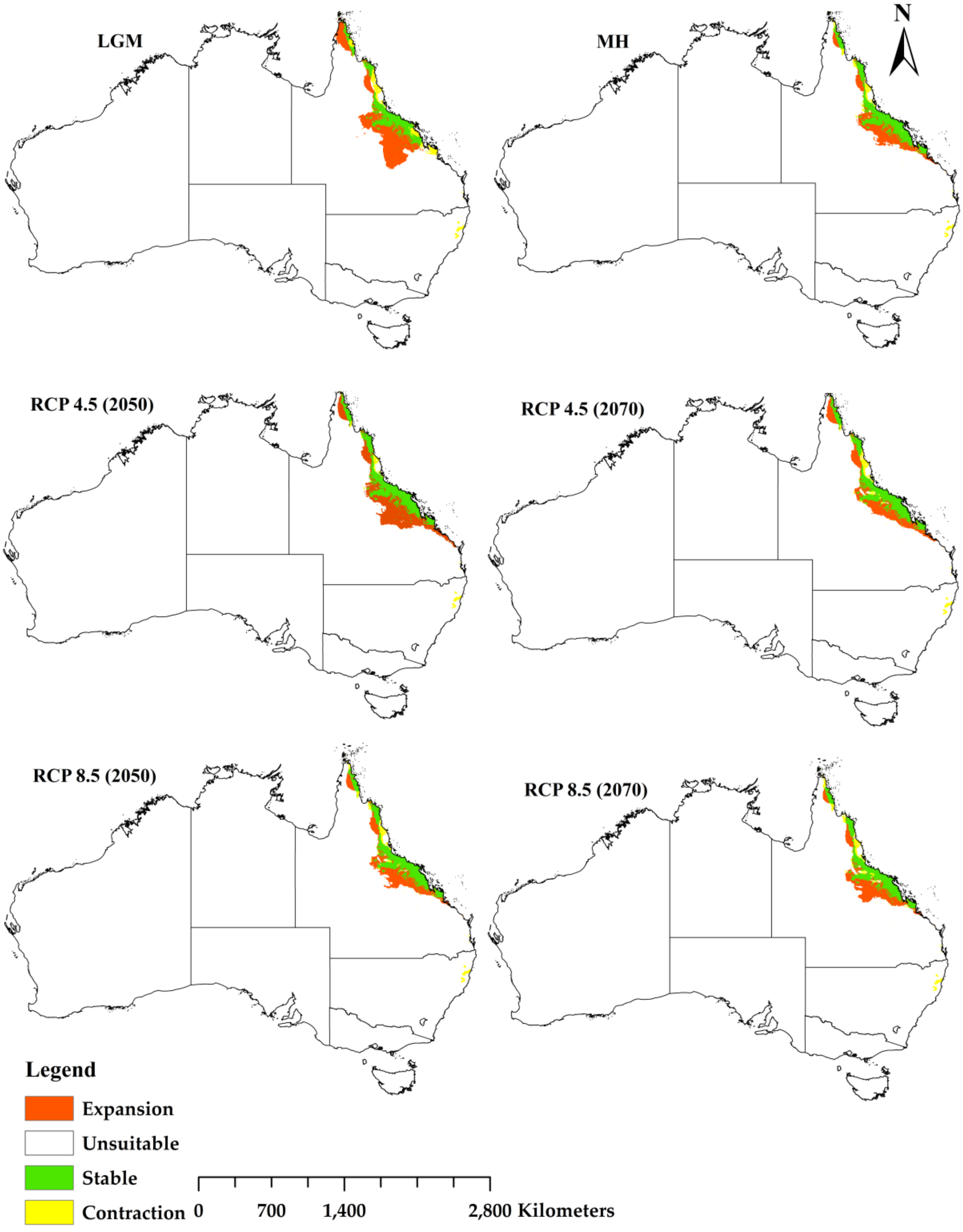

Figure S11. *N. jacobsii*.

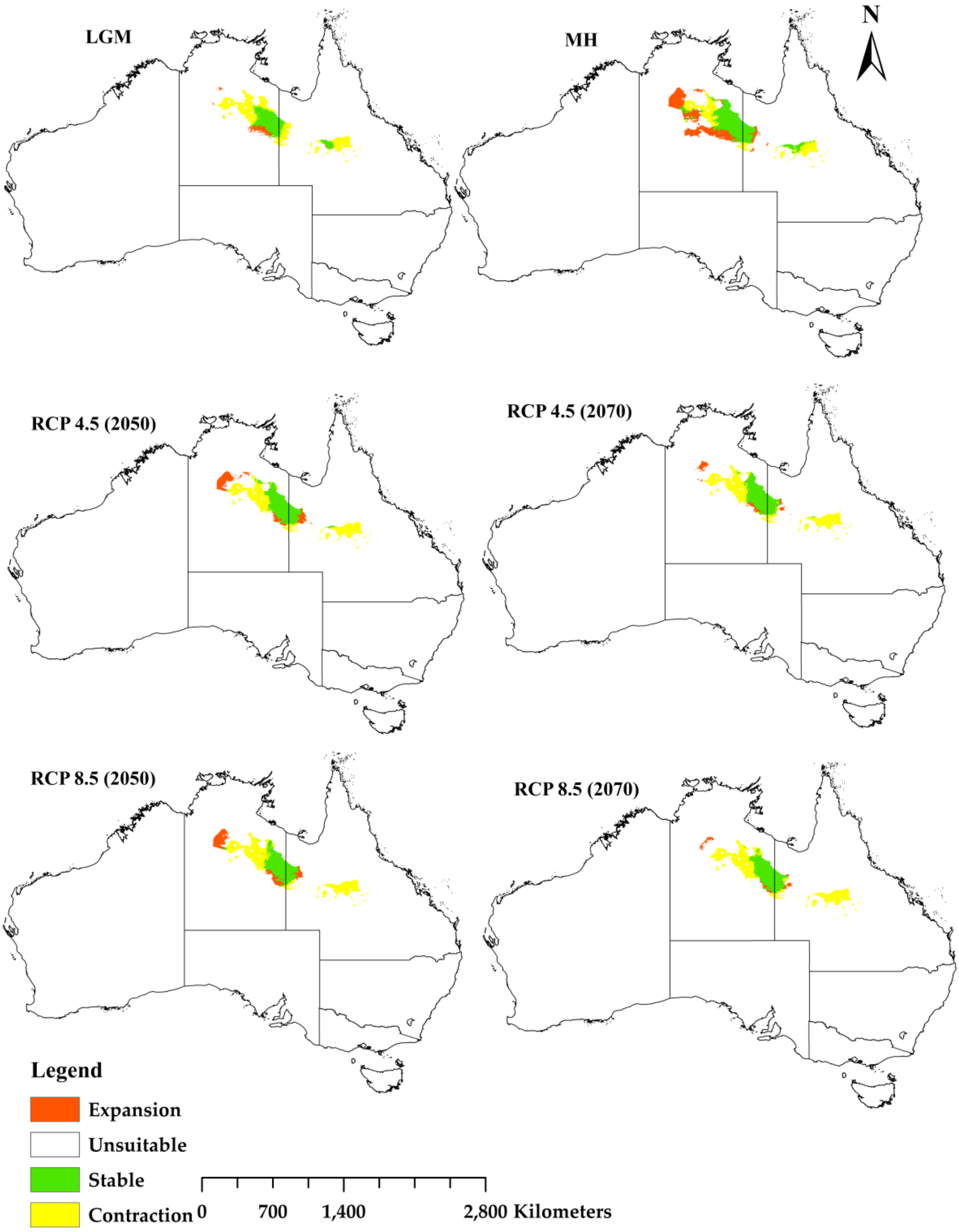

Figure S12. *N. georginae*.

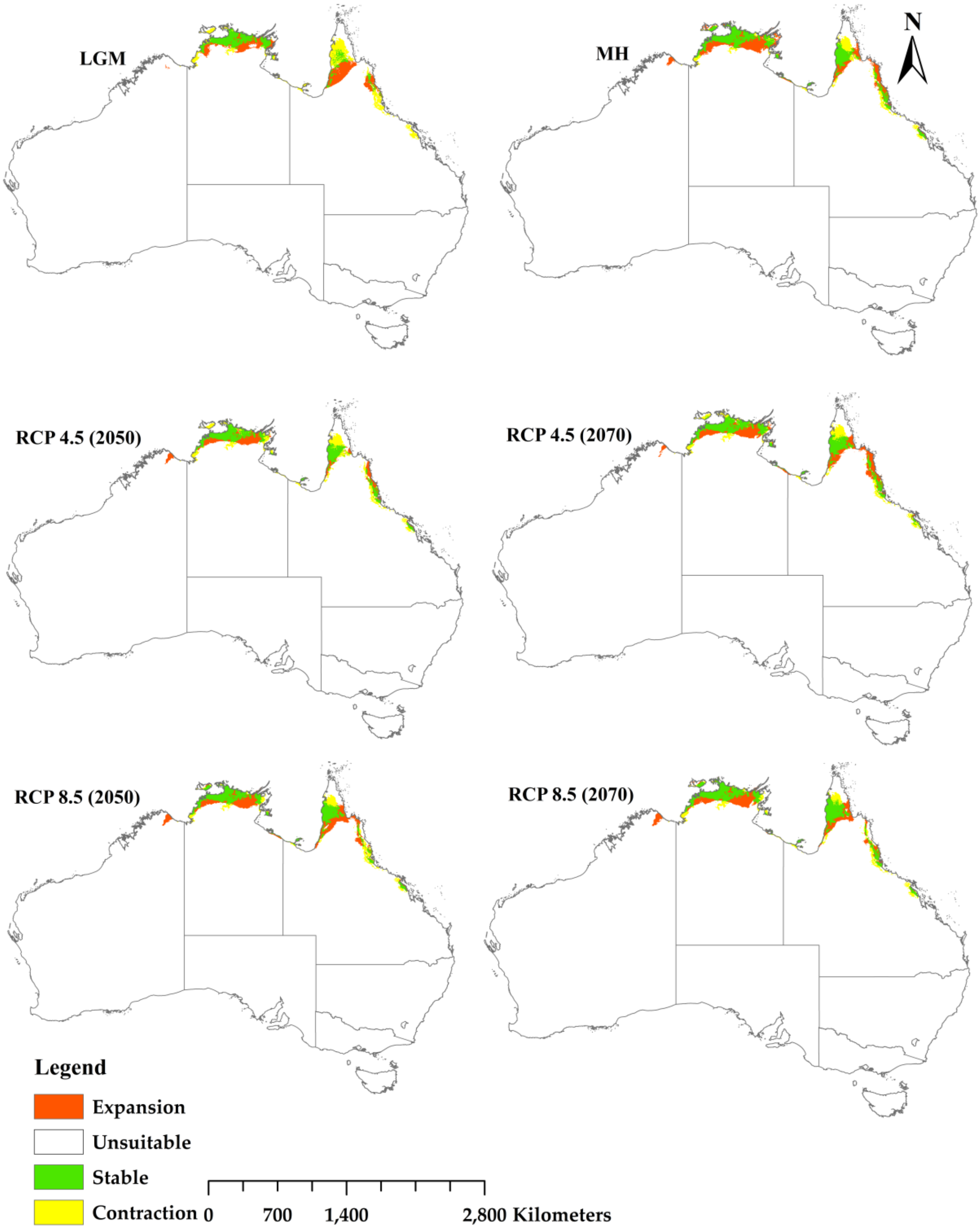

Figure S13. *N. pubescens*.

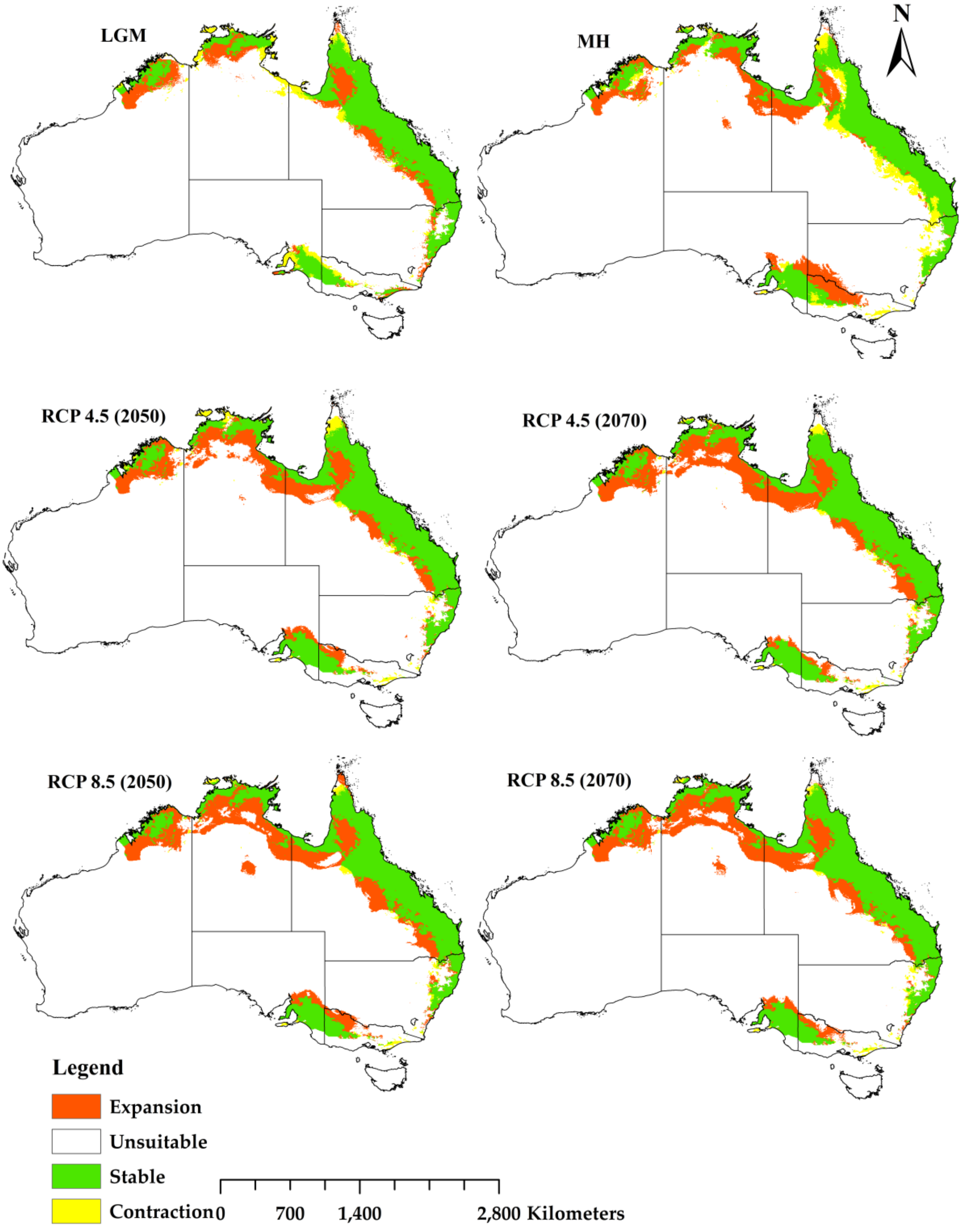

Figure S14. *N. gigantea*.
